# Temperament in Domestic Cats: A Review of Proximate Mechanisms, Methods of Assessment, Its Effects on Human—Cat Relationships, and One Welfare

**DOI:** 10.3390/ani10091516

**Published:** 2020-08-27

**Authors:** Isadora de Castro Travnik, Daiana de Souza Machado, Luana da Silva Gonçalves, Maria Camila Ceballos, Aline Cristina Sant’Anna

**Affiliations:** 1 Núcleo de Estudos em Etologia e Bem-estar Animal, Departamento de Zoologia, Universidade Federal de Juiz de Fora, Juiz de Fora, MG 36036-900, Brazil; ictravnik_cb@hotmail.com (I.d.C.T.); daianasm.dsm@gmail.com (D.d.S.M.); luana_silva1500@hotmail.com (L.d.S.G.); 2 Programa de Pós-Graduação em Comportamento e Biologia Animal, Universidade Federal de Juiz de Fora, Juiz de Fora, MG 36036-900, Brazil; 3 Department of Production Animal Health, Faculty of Veterinary Medicine, University of Calgary, Calgary, AB T3R 1J3, Canada; mariacamila.ceballos@ucalgary.ca

**Keywords:** behavior, coping styles, *Felis silvestris catus*, personality, stable individual differences

## Abstract

**Simple Summary:**

Temperament can be understood as an animal’s individuality or character. In addition, the terms ‘personality’ and ‘individual differences’ has also been reported. This article presents a literature review of more than three decades of research on temperament in domestic cats, emphasizing its origins and development, methods of assessment, and how the structure of temperament in cats was deciphered. The effects of temperament on human–animal relationships and welfare are also included. Advances in cat temperament research are presented and discussed, identifying possible gaps in knowledge, as well as opportunities for future research.

**Abstract:**

Temperament can be defined as interindividual differences in behavior that are stable over time and in different contexts. The terms ‘personality’, ‘coping styles’, and ‘behavioral syndromes’ have also been used to describe these interindividual differences. In this review, the main aspects of cat temperament research are summarized and discussed, based on 43 original research papers published between 1986 and 2020. We aimed to present current advances in cat temperament research and identify potential gaps in knowledge, as well as opportunities for future research. Proximate mechanisms, such as genetic bases of temperament, ontogenesis and developmental factors, physiological mechanisms, and relationships with morphology, were reviewed. Methods traditionally used to assess the temperament of cats might be classified based on the duration of procedures (short- vs. long-term measures) and the nature of data recordings (coding vs. rating methods). The structure of cat temperament is frequently described using a set of behavioral dimensions, primarily based on interindividual variations in cats’ responses toward humans and conspecifics (e.g., friendliness, sociability, boldness, and aggressiveness). Finally, cats’ temperaments have implications for human–animal interactions and the one welfare concept. Temperament assessment can also contribute to practical aspects, for example, the adoption of shelter cats.

## 1. Introduction

The interpretation of non-human animals having a ‘temperament’ or ‘personality’, as humans do, was debated within the scientific community until a few decades ago [1,2]. However, this scenario has substantially changed and recently, there has been a rapid increase in research on temperament in non-human animals (from this point on, referred to as ‘animals’) [3,4,5,6]. These studies have been conducted to describe the basic structure of temperament in several species and its implications in a wide range of contexts, for example, the handling of wild animals in captivity; farm animals; and companion species, mainly dogs and cats [3,4,6,7,8,9].

Although domestic cats are popular pets, the number of cat-related scientific studies is generally much lower than that of dogs [10]. Even fewer studies have been conducted on cat cognition, which is an area that has recently become of increasing interest amongst researchers [11]. Despite the increasing interest in cats’ mental functioning, there are still several unanswered questions [11,12,13]. For example, how does temperament affect their cognitive processes? Answering these questions may improve the human–cat relationship, contributing to the development of best practices for cats’ care [11]. In other words, understanding cats’ cognitive processes and temperament might contribute to the enhancement of their welfare and reduce abandonment, relinquishments, and even euthanasia that may be performed for behavioral reasons in some countries [14,15].

The existence of interconnections among companion animal welfare; human wellbeing; and the physical, social, and biological environment, is known as the one welfare concept. When applying this concept, consideration should be given to the extent to which issues can affect both companion animals and the people around them, which often extends beyond owners and their immediate environment [16].

Therefore, this literature review aims to summarize the available knowledge relating to cat temperament, addressing its proximate mechanisms (i.e., its development and genetic and physiological bases), methods of assessment, implications for the human–cat relationship, and the relation to the one welfare concept. Gaps in the existing knowledge are identified, as well as opportunities for future research. We hope to stimulate more studies in this research field that will increase the basic understanding of the mental functioning of this species and improve the quality of life for cats and their owners.

This is not the first literature review focused on temperament in cats. Previous review articles have been published by Gartner and Weiss [17], who looked at temperament in felid species in general, and Gartner [4], who studied temperament in companion animals, including domestic dogs and cats. However, this is the first extensive literature review exclusively focused on temperament in domestic cats.

## 2. Literature Search

When searching for articles, we followed the same procedures as Gartner and Weiss [17]. Briefly, we used the terms ‘cat’ AND ‘temperament’, and ‘cat’ AND ‘personality’, in the PsychInfo, CAB Abstracts, and Web of Science databases. The results were primarily selected based on the title, and later on the abstract and keywords. Original research articles published in English in peer-reviewed scientific journals were included. Academic theses, abstracts, book chapters, and review articles were excluded. In the few cases in which doubts arose regarding the suitability of a specific article (e.g., studies addressing some specific aspects or dimensions), we evaluated whether the authors discussed their findings in terms of personality, temperament, stable individual differences, or coping styles. The inclusion of papers was checked by two authors independently (A.C.S. and I.C.T.). Additional articles not captured in the search were included when cited in the reference list of Gartner and Weiss [17] and Gartner [4], and if they met the predefined criteria described above. In total, 43 original research articles were included in this review (Table 1).

Between 1986 and 2013, a total of 17 original research papers about cat temperament were published, according to Gartner and Weiss [17], whereas an update by Gartner in 2015 resulted in an increase to 24 papers [4]. A more pronounced increase was observed from 2015 to 2020, with an increase from 24 to the 43 articles included in the present study, which follows an evident trend of expansion in investigations about animal temperament/personality. Despite the new findings, the present review will differ from the two previous reviews conducted by Gartner and Weiss [17] and Gartner [4], because the present review explored causal factors and the temperament structure in more detail, whereas the previous reviews emphasized the methodological aspects of method reliability, inter-observer reliability, internal consistency, and test-retest reliability. Since the same types of methods were used in the added papers, and the results regarding the validity and reliability of existing methods have previously been presented, we will not describe these in detail again in the present review.

## 3. What Is Temperament?

For human research, temperament and personality each have their own definition. However, in animal research, few authors have distinguished between these two terms [18,19], and in most of the literature about individual differences in animals, these two terms have been used interchangeably [20,21]. They have been defined as interindividual differences in behavior, stable over time, and in distinct contexts [22,23,24,25]. The context refers to any external stimulus or environmental condition in which the animal is exposed in the moment of behavioral expression [23]. Terms other than temperament and personality have been used, such as coping styles and behavioral syndrome, with subtle variations in definitions, but converging to stable interindividual differences in behavior. In the present review, we will use the word temperament. The term coping styles is used to express interindividual differences in behavioral and physiological responses to a potential stressor, and to distinguish between different styles of responses [26]. Individuals defined as proactive copers would react offensively/aggressively to a potential stressor, whilst those defined as reactive copers would react more flexibly and defensively [26], comparable to bold and shy styles, respectively [27]. In turn, the term behavioral syndrome is more frequently used in the fields of evolution and behavioral ecology to express a set of correlated traits that tend to be stable over time, when addressing questions related to their costs and benefits to fitness [28].

Stable individual differences in behavior comprise a set of temperament traits, which are combined in main dimensions that define the structure of temperament in a given species [29]. Réale and collaborators [25] exemplified five main dimensions of animal temperament as follows: Shyness-boldness, expressing the reaction to any situation involving potential risks, such as the reaction to predators or humans; exploration-avoidance as the reaction to a novel situation, for example, a novel environment or food; activity as stable differences in the general level of activity, assessed in non-risky and non-novel environments (in animals’ usual routines); sociability, expressed in social contexts, such as reactions to the presence (or absence) of conspecifics, except for agonism; and aggressiveness which is also shown in social contexts, but specifically refers to agonistic responses [25]. An animal’s individuality results from the combination of these multiple dimensions, established by the tendencies of a higher or lower predisposition to express each temperament trait.

Most studies of animals have focused on describing temperament traits and dimensions in various species from the perspective of behavioral science and its connections with comparative psychology, evolution, genetics, physiology, ecology, and anthrozoology. Primates were the first subjects to be studied, given their phylogenetic proximity to humans [30,31]. Subsequently, the variety of species expanded, and farm [7,32] and companion animals [33,34] were also subjects of studies about behavioral individual differences. Temperament studies with wild animals have mainly been conducted among captive specimens, for a wide range of vertebrates, including fish [35,36], reptiles [37], felines [38], birds [39,40,41], and mammals such as rhinoceros [42] and elephants [43,44], in addition to invertebrates. For the latter, some dimensions comparable to those of vertebrates were reported, e.g., boldness, aggression, and activity, among others. In invertebrate species, e.g., *Caenorhabdita* worms, octopus, *Euprymna* squids, spiders, *Gryllus* crickets, *Polistes* wasps, and ants, stable behavioral patterns were reported [45,46]. Therefore, it is possible to infer that temperament has the potential to be investigated from a comparative and evolutionary perspective to identify the selective pressures that shaped its traits [47].

The fitness benefits of a given temperament type might be ‘frequency-dependent’ in a population, showing that temperament can be adaptive, modulated by genetic mechanisms and the effects of the same evolutionary mechanisms that shaped the morphology and behaviors of animals [22]. In domestic cats, there is a lack of studies about temperament addressing ultimate or evolutionary questions, such as its adaptive significance or phylogenetic comparisons. We identified a single cross-species study with a comparative approach in felid species, including domestic cats [38]. Gartner and collaborators [38] have contributed to a discussion about the evolutionary history of temperament in the Felidae family, suggesting possible fitness benefits of temperament traits in the studied species. Although the authors inferred an adaptative nature of felids’ temperament, proposing that the overall personality structure might have evolved early in felid species, this assumption lacks scientific support [38].

## 4. What Are the Proximate Mechanisms of Cats’ Temperament?

### 4.1. The Genetic Basis of Cats’ Temperament

Temperament is a phenotypic trait influenced by genetics, the environment, and their interactions. Environmental effects begin during in utero development and might last longer, affecting temperament by the experiences accumulated throughout an animal’s life [48].

The genetic bases of temperament in cats have received little attention [49,50,51], compared to other aspects, such as behavioral and physiological issues. These studies began in the 1980s and 1990s by investigating the paternal effects on kittens’ temperament traits, and demonstrated the existence of a paternal genetic influence. Studies demonstrated a paternal effect on the dimension of friendliness towards humans, revealing that part of the variability of the response of kittens to people was due to the fathers’ temperament [52,53]. The offspring of friendly fathers had a lower latency to approach and touch a human observer and a novel object, and remained closer to these stimuli for a longer interval, which could indicate a relationship between friendliness and boldness [20].

Only one study has estimated the heritability and genetic correlations of temperament traits (activity level, contact with people, aggression towards strangers, aggression towards family members, aggression towards other cats, shyness towards novel objects, and shyness towards strangers) in Ragdoll, Maine Coon, and Turkish Van cats [51]. The heritability was moderate, ranging from 0.40 (shyness traits) to 0.53 (aggression traits), and genetic correlations were also moderate, ranging from −0.32 to 0.63 [51]. In the same study, the authors assessed the effect of breed on aggressiveness, extroversion, and shyness; they reported that British Shorthair, Norwegian Forest, Ragdoll, Persian, and Saint Birman cats were less aggressive, extroverted, and fearful than other breeds [51]. In contrast, Bengal and Russian Blue breeds were more fearful and extroverted, whereas Turkish Van and Angora were more aggressive [51]. Korat breed cats were regarded as sociable, as they had a higher frequency of seeking contact with people, whereas British Shorthair cats did not have this trait [51]. Breeds also differed in terms of activity; the most active breeds were Cornish Rex, Korat, and Bengal, whereas British Shorthair, Ragdoll, and Saint Birman breeds were less active [51].

Genetic polymorphisms in the oxytocin receptor gene (OXTR) have been shown to be associated with the temperament dimensions of roughness [50] and friendliness [49] in cats. Clearly, there are vast possibilities for further research that can elucidate the genetic structure underlying the variability of temperament traits in cats. Such approaches could also contribute to cross-species molecular and genomic studies, in order to reveal the evolutionary tendencies of temperament in this species based on molecular biology and genomics tools. For example, genome-wide association studies (GWAS) would identify the genomic regions responsible for major contributions for temperament dimensions in domestic cats, as reported for other species [54].

### 4.2. Development of Cats’ Temperament

The development of cats’ temperaments is dependent on genetics and environmental effects. The latter can be characterized as pre-natal (e.g., maternal stress and nutritional and endocrine conditions to which the fetus is exposed in utero), and post-natal effects (e.g., litter size, position in the litter, competition for resources, etc.) [55]. Individual differences could be identified early in the life of kittens. The frequency of emissions of separation calls by kittens during brief separation experiments was stable from the third day to the third week of life [24,56]. Therefore, they could be used as an early predictor of cats’ temperaments in infancy, as individuals that vocalized more were more anxious in the first weeks of life than those with a lower frequency of vocalizations, considered to be calmer [24,56]. It remains unknown whether interindividual differences in separation calls and motor activity, apparently stable in the early infancy of kittens, could be useful for predicting temperament in adulthood [24,56,57]. This hypothesis was not tested in these previous studies, and the question remains open for future investigations.

Likewise, individual differences in the behavioral responses to humans were, in part, attributed to kittens’ early socialization with humans during the sensitive period of life [20,58]. Kittens that went through a process of socialization between the second and 12th week of age were more friendly to familiar and unfamiliar humans, compared to kittens that were not socialized [20]. Furthermore, Lowe and Bradshaw [59] reported that socialization with people, conducted through positive management during the first eight weeks of kittens’ lives, influenced the boldness dimension until four months of age. These authors also suggested that temperament dimensions can be stable from the fourth month to the first two years of age [59].

The long-lasting environmental effects occurring early in a kitten’s life are not well-understood [23]. Information about long-term effects on temperament have mainly be derived from studies with laboratory animals (e.g., rats). In those studies, early developmental effects, such as the body weight and litter size, affected temperament expression in adulthood [60]. Perhaps these factors also have an important role in cats’ behavior. However, confirmation of these assumptions requires long-term experiments, from infancy to maturity, which have not been reported for most temperament traits.

### 4.3. Physiological Mechanisms

Research has addressed the physiological mechanisms underlying the behavioral variation in cats’ temperament, investigating neural and neuroendocrine functioning associated with behavioral characteristics. Regarding the neural basis, distinct functional regions affect some temperament traits or dimensions [61,62]. For instance, two anatomically distinct areas in the hypothalamus were associated with the control of two types of aggressive responses in cats, indicating trait-specific neural control [61]. One controls defensive aggression (e.g., threatening vocalizations and postures, and strikes upon provocation), whereas the other controls aggression expressed by predatory conduct (e.g., silent predatory attacks, killing prey without prior vocalization, or threatening postures) [61]. Similar results elucidating the neural mechanisms of individual differences in cats’ temperaments were obtained by Adamec [62]; they reported that variability in the activity of the amygdala, i.e., the transmission between the amygdala and the ventromedial hypothalamus, underlies defensive aggression. Mechanisms underlying defensive aggression were also related to functional differences in the serotoninergic pathway from the medial hypothalamus to the dorsolateral quadrant of the midbrain periaqueductal gray matter (PAG), contributing to our understanding of the role of serotoninergic activity in aggression [63].

Motor laterality (also known as motor bias) has also been related to emotional responses in animals. Cats with non-lateralization of neural functions were more fearful in a temperament test than those classified as having left- or right-paw preference [64]. In summary, neural mechanisms linked to stable emotional responses of aggressiveness and fear are best supported by the literature, but little is known about the neural functioning underlying other temperament traits or dimensions.

The physiological basis of behavioral differences was assessed, with an emphasis on the simpato-adrenomedullary and neuroendocrine responses to stress, which vary as a function of temperament for several animal species [65,66]. Therefore, it would be expected that cats with higher behavioral stress reactivity, defined as the magnitude of the behavioral stress responses, also have more intense physiological responses to stress [65,66]. Such reactive cats might have higher concentrations of catecholamines and glucocorticoids in response to a potentially stressful situation than calmer cats. However, most of the literature does not support this assumption [21,67,68,69,70]. Previous studies did not identify any association between a temperament scoring system, as the Feline Temperament Profile (FTP), and cortisol concentrations in either saliva [21] or plasma [67], suggesting that the FTP was not useful for predicting adrenocortical responses in stressed cats. Similarly, temperament dimensions were not correlated with fecal cortisol in studies conducted by Ramos et al. [68], Stella and Croney [69], and Fukimoto et al. [70].

Beyond these investigations on the relationship between temperament and the intensity of physiological responses to stress, studies have also tested whether the styles of responses to stress vary as a function of temperament [26]. The authors [26] expected that animals with a proactive coping style (comparable to the terms active or bold) would display higher motor activity, such as exploration and escape attempts under stressful situations, along with low hypothalamus-pituitary-adrenal (HPA) axis reactivity and high sympathetic reactivity [26,65,66]. In contrast, reactive copers (comparable to passive or shy styles) would have low motor activity, along with high HPA axis and parasympathetic reactivity and low sympathetic reactivity [26,65,66]. The results of Marchei et al. [71] partially confirmed these predictions. According to these authors, there were two diverging behavioral and physiological styles of responses to stress in kittens, comparable to proactive and reactive copers, when exposed to an open field and a novel object, or aversive stimulus tests. However, the two coping styles did not differ in terms of the physiological parameters measured. Reactive animals were expected to present bradycardia induced by a predominance of the parasympathetic system, in response to a sudden unpredicted stressor. However, emotional tachycardia was observed in animals of both styles, leading the authors to suggest that sympathetic system activation in cats might differ from that of other species [71]. Therefore, additional research comparing domestic cat temperament styles and their underlying physiological responses is needed, preferably using minimally invasive physiological indicators.

The surface body temperature has been proposed as a practical and non-invasive indicator of the physiological response to acute stressors [72]. Foster and Ijichi [72] reported a negative correlation between the ocular temperature and FTP scores in cats, suggesting that animals with higher ocular temperatures (i.e., more intense responses to stress) had lower frequencies of acceptable responses in FTP. Therefore, cats with excitable temperaments might be more sensitive to stress, as measured by the eye temperature.

### 4.4. Correlations with Other Phenotypical Traits

There is a belief that cats with specific coat colors are more likely to have an excitable temperament. Moreover, it is also known that selection for tameness might lead to correlated changes in phenotypic traits, e.g., coat color pattern, ears, and tail morphology, known as the domestication syndrome [73]. Therefore, correlations between cats’ temperament and morphology, e.g., coat color, were assessed, based on the assumption that genetic linkages, such as pleiotropy, might explain them [74].

In one study aiming to assess the relationship between polymorphism in coat color patterns and life-history traits in cats living in rural and urban areas, the allele coding for an orange coat was related to the tendency for a higher aggressiveness [74]. According to the authors, cats with an orange allele would be more involved in fights, increasing the risk of death and reducing the chances of reproductive success in urban environments; this accounted for the lower frequency of the orange allele in tom cats living in urban, compared to rural, areas [74]. Additionally, Stelow et al. [75] suggested that the relationships between coat color and temperament might be sex-dependent. Orange females (tortoiseshells, calicos, and ‘torbies’) were reported to be more aggressive towards people than any of the other colors investigated, based on the owners’ perceptions. In contrast, black, white, and gray and white tom cats were characterized as more aggressive than other colors [75]. Although some patterns were revealed, the authors concluded that these differences could also have been due to owner bias.

In fact, Delgado and collaborators [76] reported that owners might attribute certain temperament traits to cats based on their coat colors; therefore, coat color could be a source of bias and not a real attribute of cats’ temperament. Owners perceived orange cats as more friendly and less shy than white and tortoiseshell colors, which were perceived as more indifferent, intolerant, and less friendly. Although owners had attributed certain traits as being influenced by coat color, they did not rule out that some biological basis might exist and warrant further investigation [76].

Wilhelmy et al. [77] subsequently proposed that the relationships between behavioral traits and patterns of coat and eye colors were dependent on breed, given the low occurrence of associations independent of the breed effect [77]. The authors proposed that there was a low level of pleiotropy between these traits [77]. In summary, available scientific evidence does not unquestionably support that any physical characteristic of cats is a reliable predictor of temperament, but also does not definitely eliminate this possibility [75,76,77]. Investigations conducted to date have used owners’ reports to characterize cats’ temperament profiles. Therefore, for future research, objective methods of assessment based on behavioral observations and evidence at the molecular level, such as pleiotropy and/or linkage disequilibrium, would better elucidate the mechanisms behind the hypothetical temperament and morphology relationships.

## 5. How Has Temperament Been Assessed in Cats?

Temperament assessment in cats has taken into account animals’ behaviors and emotional expressions that are consistent across time and in different contexts [9,21,34]. According to the widely accepted concept of temperament, consistency is a fundamental condition for a given behavior to be considered a temperament trait [23,25]. Therefore, in terms of assessment, measurements are commonly conducted multiple times with the same individual and/or various tests are applied [21,78,79]. A set of temperament measures have frequently been used [80].

Methods used to assess cats’ temperament can be categorized, according to the duration of procedures, as short- vs. long-term measures. In addition, according to the nature of recordings, methods can be classified as coding vs. rating methods. Temperament indicators are also combined with pre-established measurements and procedures in assessment protocols. These various approaches are described below.

### 5.1. Short- vs. Long-Term Observations

According to the duration of behavioral observations, temperament assessment can be conducted using short- or long-term measures. For the former, animals have been exposed to momentaneous situations (or stimuli), through standardized behavioral tests in controlled environmental conditions (i.e., experimental settings) [26,79]. In most of these studies, tests lasted from 1 to 10 min [20,79,80]. Although situations that elicit positive mental states could be used [80], most of the temperament studies involved tests with potentially stressful or challenging situations, such as physical restraint, separation, or an unpleasant stimulus, e.g., a spray bath [67,69,79]. The goal of these studies was to reveal individual emotional and behavioral variability by exposing cats to brief situations that create a certain pressure. When these variations are stable over several trials, then they are considered to be a temperament trait [79]. Before the behavioral recording begins, animals are usually allowed to habituate to the experimental setting for 1 to 30 min [20,67,80].

Here, we present some examples of behavioral tests traditionally used for temperament assessment, applied in studies with domestic cats:

- Responses to humans [20,69,80]: This approach is used to assess the animals’ responses towards a familiar or unfamiliar person, or both, depending on the aim of the study [20,69,80]. When the test is used to reveal fearfulness toward humans, an unfamiliar person must be chosen [81]. Although the test procedures might vary, the observer usually begins the test stationary, ignoring the animal. In subsequent phases, the observer approaches the cat and tries to have physical contact (touch, hold the cat against the observer’s chest, stroke, or pull the cat’s tail). There are several potential measures, e.g., the cat’s latency to approach, its voluntary approach towards the observer, the flight distance (defined as the maximum distance the cat enables the observer to approach before withdrawing), the latency to interact, and/or the duration of the interaction, among other behaviors (body posture, ears and tail position, inactivity, locomotion, approach, vocalizations, kneading the paws, physical contact, rubbing the person, drooling, eye contact, and play) [20,69,80,81];
- Novel object test [20,78]: This is used to assess responses towards novelty (from neophobia to neophilia). A novel object is placed in a test arena, or in the cat’s home pen or a familiar environment, in order to assess the behavioral responses towards the object. The existing studies diverge about which type of object to use, leading to divergent results. For instance, in the study of Finkler and Terkel [82], the authors used a cage containing a live mole-rat (*Spalax ehrenbergi*) as a novel object. One could expect that a live animal might elicit reactions distinct from those elicited by an inanimate object. For example, a live animal would elicit predation behaviors and predatory aggression, whereas a novel object would be more related to exploration, curiosity, and reaction to novelty. Therefore, it could be helpful to standardize the use of inanimate objects. According to Durr and Smith [78], the object must combine two desirable features: (i) Movement to cause attraction and (ii) moderate to high noise to elicit hesitation. They recommend avoiding objects that are too intense or frightening and also those that are too neutral or uninteresting [78]. The behaviors recorded are usually the distance to the object, latency to approach, visual contact, and tactile interactions with the object;
- Food offering test [82,83]: This is used to assess cats’ responses while receiving food offered by a human observer. Anticipatory behavior, anxiety, and reactions to humans are some of the aspects studied. First, the observer holds the food in the animal’s presence and, in the sequence, the food is offered and recording is finished when the cat accepts the food (i.e., starts eating). Post-meal behaviors might also be recorded. This test can be used with animals kept individually, or in a group, in order to assess the dimensions of food dominance [82] and sociability [83];
- Open field test [71,81]: This is traditionally used to assess anxiety, which might be more or less intense as a function of individual temperament [81]. Animals are exposed singly in a test arena or an unfamiliar closed and empty room. Motor responses (activity vs. inactivity) and vocalizations, as well as other behaviors of interest, are recorded;
- Test of confinement [79]: This is used to assess the level of behavioral reactivity of cats when exposed to a situation that involves physical restraint and isolation. A box/carrier for transportation can be used. Behaviors expressing motor responses and vocalizations are usually recorded;
- Stress test [67]: This is used to assess cats’ styles of responses towards a short-term stressor, which elicits acute stress responses, for example, the spatial restriction of the cat in a cage and a 3-min spray bath. Motor responses and vocalizations are recorded. Iki et al. [67] proposed that this type of test might be used to identify cats’ coping styles (styles of stress reactivity). A higher motor response indicates a proactive (active) coping style, whereas cats that vocalize more frequently indicate a reactive (passive) coping style [67]. However, this kind of test may have welfare and ethical issues related to the intensity and duration of the stressor used. Therefore, the definition of a kind of ‘behavioral endpoint’ is recommended, in which behavioral-based criteria are used to interrupt the test if the animal has extreme reactions, indicative of panic responses.

Temperament can also be evaluated through long-term measures, monitoring individuals for several days in their daily routine, during their habitual life conditions [27,34]. By monitoring cats’ behaviors for relatively long intervals, they may be more likely to express behavioral patterns reflecting their individuality [69]. Long-term measurements have the potential to reveal aspects or dimensions that behavioral tests do not assess, for example, the general level of activity and spatial movement patterns within the home range [25,84]. Behavioral recordings use an ethogram to address the behavioral repertoire over several days of observations [27]. Alternatively, long-term measures can be based on information provided by cat owners or caretakers, using the familiarity and knowledge accumulated by the owner throughout the whole time of interaction with the animal [51,69]. For example, Feaver et al. [34] assessed the temperament of cats using observed behavioral categories (e.g., sniffing, looking, playing, eating, sleeping, and others) recorded over 15 consecutive days (a long-term coding method). After three months of contact with the animals, the assessors rated cats’ behaviors based on the familiarity and knowledge they gained about the animals, rating temperament in a list with 18 adjectives, such as aggressive, curious, or excitable (a long-term rating method).

### 5.2. Coding vs. Rating Methods

Regarding the nature of the measurement (i.e., type of behavioral recording), temperament assessment methods can be categorized as coding (or quantitative methods) and rating (or qualitative methods) approaches [69,85,86]. Among the 43 studies included in this literature review, 53.49% (23/43) used rating methods, 39.53% (17/43) used coding methods, and only 6.98% (3/43) combined both types of methodologies (Table 1).

Coding is based on the quantitative recording of behaviors through direct observation, recording the frequencies and durations of the sampled behavioral categories [27,69]. The sampling unit is the individual, and the most common sampling rule is focal sampling, in which a target individual is observed for a given interval, recording all behaviors of interest [87]. Even when the behavior is recorded in groups, for example, to assess the dimension of sociability or dominance, individual identification is required, enabling the observer to perform a quantitative recording per individual [27,78]. One limitation of this method is the time and effort required to quantify several behavioral categories for each studied animal. However, the coding record has the advantage of being objective, allowing automated behavioral recordings and/or the use of software that could facilitate data collection [88].

Rating methods are characterized by describing the animals’ emotional states and body language through observers’ perceptions. Lists of adjectives may be used as predefined descriptors of temperament (e.g., aggressive, fearful, irritated, calm, relaxed, active, and nervous), quantified on a visual analog scale or Likert scale [2,86,89,90,91]. This method is widely used to assess cats’ temperament, employing descriptor adjectives quantified using Likert scales ranging from 1 (‘*not at all*’ or ‘*does not describe my cat*’) to 5-, 7-, or 9-points (‘*very much so*’ or ‘*describes my cat extremely well*’) [19,34,38,49,50,51,69,89,92,93,94,95,96].

The adjectives can be followed by a brief, behavior-based definition to enhance their comprehension and consistency of use between observers, as in Litchfield et al.’s [9] study, in which the authors defined each adjective, for example, “*vigilant = watchful or observant; spends a lot of time attending to his/her surroundings; stable = reacts to his/her environment in a calm way; and bold = daring, not restrained or tentative, doesn’t hesitate*”. There is also the option to use questionnaires with short sentences to classify the behavioral responses employing scales that range from ‘strongly agree’ to ‘strongly disagree’, for example, ‘*my cat challenges the usual dominance order with other cats/people in the household*’ [8]. Although questionnaires of defined adjectives or sentences are considered more understandable by some authors [8,9], in most studies, the adjectives are used without any formal definition. If compared to the sentences, adjectives are usually considered more advantageous for being extendable to a wide range of species, leading to fewer concerns related to translation into other languages, in addition to generating more concise questionnaires [89]. Long questionnaires are time consuming and hamper the voluntary participation of respondents in the research [89]. Usually, tens of adjectives are used, with diverse meanings that express emotional states of positive, negative, or neutral valence. There is considerable variation in which adjectives are used in cat temperament studies (Supplementary Material Table S1).

According to Meagher [97], the main advantage of observers’ ratings is their integrative character, allowing them to gather several traits on a single scale or dimension. The ratings are also more practical and less time consuming than coding methods and do not require previous training or expertise in behavioral observations [57]. Since this method does not require vast experience from the observer, rating scales enable the use of the knowledge and familiarity of owners or caretakers to evaluate their animals [19]. These characteristics allow a considerable increase in the sample size of temperament studies using rating methods [89]. The average sample size in studies with rating methods was 688.65 (range, 8 to 5726), whereas for coding studies, it was 40.35 (12 to 139), and for studies using both, it was 43.67 (14 to 62) (Table 1).

The main limitation of the rating methods is the higher risk of bias, leading to results not reflecting the real attributes of animals’ temperament, but a biased judgment of the observer [94]. Some features affected the observer’s ability to detect subtle aspects of cats’ body language, including professional experience, the attachment (or bond) with the animal, and previous experiences with the assessed species [98,99]. Cats’ physical aspects could also be a source of bias, as previously mentioned [76]. Besides, there is also a tendency for female respondents to be more willing to participate in questionnaire studies [8,9,19,76,93,94], leading to a possible gender bias in the temperament evaluation, as previously reported [93]. Finally, observers’ ratings have been criticized on the basis of the level of anthropomorphism that is inherent to the method, defined as the attribution of human motivations, characteristics, or emotions to non-human animals [93]. There are only a few studies that have compared the outcomes of rating and coding methods and they concluded that they are, to a certain extent, in agreement [34,69,88].

### 5.3. Protocols of Temperament Assessment

Some initiatives have been implemented to develop standardized protocols of temperament assessment in domestic cats:

- The Feline Temperament Profile (FTP) is a questionnaire-based approach focused on assessing cats’ reactions to humans [21,64,67]. The FTP has been considered valid and consistent over time. Furthermore, it has been regarded as useful for predicting cats’ stress responsiveness, given the correlations with behaviors obtained in situations that did not involve human contact [21]. The FTP is composed of 10 situations representing a rising level of stimulus by contact with a non-familiar observer: ‘Initial approach’; ‘follow up approach’; ‘friendliness’; ‘interaction’; ‘play initiation’; ‘sociability I’; ‘sociability II’; ‘adaptability’; ‘aggressiveness or fear I’; and ‘aggressiveness or fear II’. In each phase, cats are categorized based on a behavior-based predefined score of two points (acceptable vs. questionable behavior);
- The Cat Tracker Project Questionnaire is a rating temperament assessment protocol that uses a questionnaire for cat owners [8,9]. Temperament is recorded based on five main dimensions, named as ‘The Feline Five’ [9]. An example of an item is ‘*My cat challenges the usual dominance order with other cats/people in the household*’ [8]. The responses are rated on a 5-point Likert scale, from 1 (‘*does not describe my cat*’) to 5 (‘*describes my cat extremely well*’);
- The Meet Your Match^®^ Feline-ality™ protocol was developed by ASPCA^®^ for cats’ personality assessment in shelters [100]. The protocol allows cats’ profiles to be separated into two main dimensions equivalent to the shy-bold axis and friendliness to humans, which have been named the ‘Valiance scale’ and ‘Gregariousness scale’, respectively. Cats are exposed to a sequence of tests composed of 11 steps: ‘Body posture’; ‘greeting approach’; ‘cage condition’; ‘social response when door is opened’; ‘introduction to novel space’; ‘call and approach’; ‘open hand’; ‘stroking’; ‘play’; ‘hug’; and ‘sensitivity’. The recording is done by assigning behavior-based predefined scores that correspond to a scoring system used to define the cat’s profile. The Meet Your Match^®^ Feline-ality™ protocol was developed for use with shelter cats, aiming to promote a perfect match between the cat and potential adopter. Adopters have to complete a questionnaire to reveal their personality [100]. The protocol has been used in studies aiming to test its effectiveness in revealing cats’ temperament aspects related to the success of adoption in shelters [70,101,102];
- Domestic Cat Personality Inventory—edition 1 (DCPI—e1) is a questionnaire composed of 29 adjectives. The DCPI is a standardized and simple methodology that can be used at the research level, allowing users to develop extensive temperament inventories based on owners’ perceptions and ratings [94].

## 6. What Do We Know about the Structure of Temperament in Cats?

The first published paper about temperament in cats was written by Feaver et al. [34], who, in 1986, described the three main dimensions of cat temperament: Alert, sociable, and equable. Since then, over the course of more than three decades, other researchers have attempted to reveal the structure of cat temperament, using a diverse array of terminologies. In the 43 studies reviewed, there were a total of 49 dimension names (Table 1).

Some papers described the temperament based on stable individual differences in one specific behavior, such as post-feeding behaviors [83], social structure [78], separation calls [24,56,79], motor activity [24,57,79], hunting behavior and prey preference [103], and the lateral bias scores [64]. Most research reported the temperament based on its main dimensions, formed by a number of correlated behavioral traits (or facets). Only six of these studies described temperament based on only a single dimension (boldness [20,27,59], sociability [80], fearfulness [81], and friendliness [52]). The multidimensional approach was widely used to describe the temperament of cats; some of the dimensions described are detailed below:

- Friendliness reflects the styles of responses towards humans or conspecifics, and the willingness to approach and have contact with humans [52], defined by the adjectives ‘sociable/calm/friendly/adaptable/gentle’ [49,50]. Several other terms were used to express the same kinds of behavioral responses, for example, amiability was defined by the adjectives ‘cooperative/warm/peaceful/charming/faithful’ [94], whereas sociable ranged from ‘sociable with people’ to ‘fearful of people/hostile to people/tense’ [34]. Sociability was defined based on latency to contact, duration of proximity to humans, and a higher frequency of meow vocalizations [80]. Similar behaviors, ranging from erect ears and sociable to vocal and locomotion, were used to express the sociable dimension by Wedl et al. [88]. Independent-gregarious and gregariousness were also used to express dimensions comparable to friendliness [95,101]. Dimensions reflecting cats’ responses to humans are among the most frequently reported, revealing the perceived importance of human–animal relationships to the definition of the structure of cats’ temperament. It could also be a result of methodological bias, since the procedures of behavioral tests often involve responses to humans. The frequent use of tests involving reactions to humans might be due to the feasibility and/or the close proximity or contact of cats with humans when the tests were conducted;
- Aggressiveness is characterized by the way cats express agonistic behaviors and the lack of tolerance to manipulation [95], which might include aggression towards other cats, strangers, and human family members [51]. Other terms were also used, such as roughness, defined by the adjectives ‘irritable/moody/defiant/dominant/aggressive/excitable’ [49]; human aggressive, defined by the adjectives ‘aggressive/hostile-people’; and human nonsocial, described by the adjectives ‘fearful of people/solitary’ [19]. It is worth noting that in most dimensions involving aggressive reactions to humans, the responses of aggressiveness to conspecifics can be included in the same dimension, revealing the importance of the human–cat relationships for the behavior of companion animals [104];
- Boldness (also known as the shy-bold axis) was previously defined in the present review as corresponding to proactive-reactive coping styles, reflecting the styles of responses towards a challenging situation [27,51]. According to McCune [20], boldness is an important aspect of cats’ temperament, and other dimensions, such as friendliness and sociable, have, in their definitions, elements equivalent to boldness (e.g., confident). The dimension named valiance can also be considered synonymous with the shy-bold axis [101];
- Openness was defined by the adjectives ‘playful/curious/inquisitive/active/inventive’ [49,50], reflecting the reactions of animals to novel experiences or stimuli (i.e., novel places, objects, and people) [102]. In Fukimoto et al.’s [102] study, cats expressed openness based on their positive responses to introduction into a novel room;
- Active is related to motor activity and attention, defined by the adjectives ‘active/agile/watchful/curious/playful/excitable/vigilant’ [19,88]. This dimension could be comparable with playfulness, described by the adjectives ‘energic/playful/quick/mischievous/curious’ [94];
- The Feline Five approach is used to express the structure of cats’ temperament based on a five-factor model, widely accepted for human personality assessment, known as the ‘big-five’ and comprised of agreeableness, conscientiousness, extraversion, neuroticism, and openness [105]. For cats, the use of a five-factor model based on orthogonal dimensions was initially proposed by Gosling and Bonnenburg [89] and later termed the Feline Five by Litchifield et al. [9], who used the dimensions of agreeableness, dominance, extraversion, impulsiveness, and neuroticism for cats;
- Agreeableness is comparable to friendliness, ranging from ‘affectionate/friendly towards people/gentle/playful/trusting/cooperative/inquisitive’ to ‘solitary/irritable/aggressive towards people/suspicious’ [8,9]. In contrast, extraversion is defined in part by responses to people, but also by the level of activity and energy [51], ranging from ‘decisive/smart/curious/inventive/active/inquisitive/vigilante/deliberate/self-assured/persevering/bold/playful’ to ‘aimless/clumsy/quitting’ [9]. The behaviors play, sensitivity, hug, and greeting approach were also used to define extraversion [70,102];
- Impulsiveness reflects the level of predictability of behavioral responses, ranging from ‘impulsive/erratic/reckless/distractible/excitable/aimless/irritable/aggressive towards people/defiant’ to ‘predictable/constrained/gentle’ [9]. Gartner et al. [38] described impulsiveness ranging from ‘excitable/active/playful/eccentric/impulsive/distractible/reckless/erratic/jealous’ to ‘constrained/independent/predictable’. In turn, neuroticism expresses variation in cats’ emotional stability, ranging from ‘insecure/anxious/fearful of people/suspicious/shy/tense/fearful of other cats/excitable’ to ‘trusting/calm/stable/self-assured/bold/cool’ [9]. Arahori et al. [49,50] described neuroticism by the adjectives ‘vigilant/fearful/attentive/nervous/timid/anxious/cautious’;
- Dominance reflects cats’ individuality in a social context, ranging from ‘bullying/dominant/aggressive towards other cats/jealous/defiant/greedy/irritable/reckless/aggressive towards people’ to ‘submissive/friendly towards other cats/gentle’ [9,38]. Bennet et al. [94] described dominance using ‘proud/domineering/serious/independent/territorial’.

The dimensions used in the existing studies represent the main aspects forming the individuality of these animals in the methodological contexts used to date. Presumably, the diversification of tests and methodological contexts would reveal additional aspects not yet described. For instance, two studies have proposed the existence of stable individual differences in cats’ predatory habits, prey preferences [103,106], and predation behavior [103]. Although poorly understood, these individual differences may characterize a temperament dimension. They do not appear in most experiments, because the trials were conducted in situations that lacked an opportunity to express predation. The understanding of stable individual differences in predatory habits should be relevant for outdoor cats, allowing more precise and individualized estimations of the potential impacts of cats on wildlife through predation.

## 7. Does Cat Temperament Affect the Human–Cat Relationship and One Welfare?

### 7.1. Importance of Temperament in the Adoptive Process and Cat–Owner Relationship

Most of the research about cat temperament has focused on its impacts on the human–animal relationship [95,102,107], emphasizing the adoptive process and relationships between the cat and its owner [8,95,102,108]. The compatibility between cats and owners usually begins at the time of adoption, since the cats’ behavioral styles are among the factors that humans use to choose an animal to adopt [108]. Raising cats’ sociability would increase the chances of adoption, because owners usually prefer cats expressing behaviors of willingness to play and interaction with humans [108]. People also tend to attribute certain traits to cats as a function of their color, which also affects the cat’s chance of being adopted [76]. For instance, black cats may have lower chances of adoption, being judged as eccentric and negatively associated with superstitions [76,109].

An owner’s personality also has an important role in this process, as it might affect the type of cat the owner would choose to adopt and even the decision to own a cat [8]. Additionally, a higher level of owner satisfaction can be achieved when there is compatibility or complementarity between the cat’s temperament and the owner’s personality [93]. Owners with personalities described as having greater warmth were more satisfied with cats that had higher values in this dimension. The opposite was reported for dominance, in which owners characterized as more dominant reported more positive perceptions of their cats when they were defined as more submissive [93].

The relationship between cat temperament and owner personality may be shaped by various factors, including the environmental effects to which the cat is exposed as a function of the owner’s personality. Finka et al. [95] reported that owners with higher levels of neuroticism were more likely to have cats with aggressive or anxious/fearful behavioral styles, in addition to more frequent stress-linked sickness behaviors and behavioral problems. Conversely, owners with more agreeableness and conscientiousness raised cats characterized as less aggressive and aloof/avoidant. Moreover, higher owner conscientiousness was related to less anxious/fearful cats [95]. This might result from environmental effects on cats’ behaviors and emotional states, induced by the way the owners behave and treat their cats. The judgment of cat behavior by the owner might also contribute to the results, as neurotic owners may ‘misattribute’ negative traits to their cats. In contrast, more agreeable owners may perceive their animal as being more positive [95].

The compatibility between cat and owner is important for their relationship. For instance, cats with temperament traits of aggressiveness and dominance might have a lower tolerance of other animals and humans, deteriorating the human–cat relationship [8]. Cats with defensive aggression associated with anxiety tend to maintain a higher arousal state, responding defensively to subtle events, e.g., attempts to pet and play [107]. Defensive behaviors, such as hiding, flight, hissing, biting, or scratching, might reduce owner satisfaction and compromise the bond between the cat and owner, reducing the welfare of both [107].

When cats’ temperament and behavior do not meet owners’ expectations, it might degrade the human–animal bond, resulting in relinquishment, and even abandonment or euthanasia in extreme cases. When these unpleasant events occur, they bring pain and suffering for humans, but particularly for the animal [110]. In a study conducted in the United Kingdom, the owners reported a behavioral reason for 38% of cat relinquishments. Among the behavioral problems reported, 44% were aggressive events towards other cats or people, and that behavior can be caused, in part, by the cat’s temperament [111].

The exhibition of other behaviors perceived by the owner as inappropriate can also be associated with cats’ temperament. For instance, the inappropriate elimination of urine or feces is a behavioral problem frequently reported by cat owners [112,113,114]. In a study about common risk factors for urinary house soiling (the inappropriate elimination of urine), cats characterized as having a non-relaxed temperament were three times more likely to display house soiling compared to cats with a relaxed temperament [113]. In summary, the relationships between a cat’s temperament, owner’s personality, behavioral problems, and the human–animal relationship are complex, and there are difficulties in determining the causes and consequences of each factor involved.

### 7.2. Relationships between Temperament and Welfare

Temperament might affect welfare in many ways. One of them is related to the environmental and management needs that might have specificities as a function of the animal’s temperament. Fearful and anxious cats tend to seek out hiding places and escape routes when they feel threatened. For these individuals, specific types of environmental enrichment should be available, expanding the use of vertical space and hiding places, allowing them to hide and have a wider view of the environment to detect possible ‘threats’ [114]. The lack of such resources may be more harmful to the welfare of nervous/fearful cats than calm/relaxed cats. In clouded leopards (*Neofelis nebulosa*) kept in captivity, individuals classified as more fearful/tense spent more time in a hiding place, displayed more self-mutilation, and had higher fecal cortisol concentrations [115].

The owners and caretakers must be able to identify the welfare needs and behavioral individuality of cats within their care [116]. In the study conducted by Rioja-Lang et al. [117] using the Delphi methodology—a research technique based on the opinion of a panel of experts—with the participation of 14 cat welfare experts in the UK, the authors reported that among several factors evaluated, the most important cat welfare concern was the social behavior issues resulting from an inappropriate home environment. Social restriction was considered the fourth most common issue. Indeed, a study involving 547 cat owners in the U.S. identified a relationship between a better level of owners’ knowledge about cat behaviors and environmental needs, with fewer reports of behavioral problems, as well as more cat care practices, with the potential to improve cats’ welfare [118]. Therefore, by identifying the cat’s individual behavioral needs, owners and caretakers might be able to adopt best practices of care and also improve housing conditions to meet these specific needs and enhance the cat’s welfare [69,102].

One of the principal aspects of welfare is health. Temperament can be associated with susceptibility to diseases and immune function, as reported for other species [119,120]. However, little is known about the relationship between temperament and susceptibility to diseases in cats. In urban feral cats, individuals classified as bold were more vulnerable to contracting feline immunodeficiency virus (FIV), which is a disease with a high mortality rate primarily transmitted via bite wounds [27]. Bold cats had higher reproductive success, but were also more involved in disputes and aggressive interactions, putting them at a greater risk of contracting FIV than shy cats [27,121]. Some authors have also argued that the immune function of animals might vary as a function of temperament [122,123,124]. To the best of our knowledge, this assumption has not been tested in cats.

### 7.3. Cats’ Temperament and Their Potential Impact on Wildlife

Connections between humans and animals must be considered within the concepts of one health, one welfare, and one biology. Despite the existence of specific differences between species and individuals, the one biology concept means that the biological principles for humans and all other species are the same [125]. Investigating biology means studying humans, together with all other species, and principles such as the environment, ecology, and conservation should not be considered in different ways concerning humans and other species [125]. The one welfare and one health concepts are complementary, where human and animal health and welfare are interconnected with the environment [126]. All actions influencing the relationship between humans, other animals, and the environment should recognize all biological aspects of all living beings [125]. Therefore, our responsibilities regarding cats need to consider their interactions with the natural environment and the balance of biodiversity.

Domestic cats’ expression of predatory behaviors varies according to the geographic region and availability of feed resources [127,128]. Predatory behavior might also show stable individual differences, as described in domestic cats, in which the willingness to hunt and the ability to discriminate numerosity and the size of potential prey have interindividual variation [103]. If some cats prefer a specific prey type, it would be reasonable to suppose that they would seek that prey, regardless of its abundance. This behavior could be characterized as a threat for prey populations with a small number of individuals or that are in decline, increasing the risks of local extinctions and loss of biodiversity in sensitive environments, such as islands [103,129]. It has been suggested that bold, aggressive, and dominant cats have a larger home range than shy and submissive ones [84,130]. As the impacts on wildlife can be proportional to the home range size, bold individuals should have more potential impacts than shy cats, which occupy smaller areas [84,130].

A better understanding of the level of flexibility or consistency in the predatory behaviors of cats is necessary. It is also crucial to understand the genetic, behavioral, ontological, physiological, and environmental factors affecting cats’ willingness to hunt and prey preferences. This information would contribute to the development of cat-friendly conservation strategies of a wide range of wild species, such as small mammals, reptiles, birds, and invertebrates, threatened by inadequate management or irresponsible cat ownership [129]. The mitigation of impacts on wildlife would reduce the aversion of some people to domestic cats, bringing indirect benefits to cats’ safety and welfare. Another factor influencing the one welfare concept is irresponsible cat ownership. Not being aware of what the cat is doing and where it is, when allowing them outdoors, not only negatively influences the environment and biodiversity, as stated before, but also the cat and owner. For example, free-roaming cats can have a higher risk of developing toxoplasmosis, which is a zoonotic disease caused by a zoonotic protozoan parasite that puts the owners’ health at risk [131].

## 8. Final Considerations

Due to over three decades of research, considerable advances have been made in the description of the structure of temperament in domestic cats, through various methods. However, there are aspects not yet fully understood; for example, the stable interindividual variation in predatory behaviors. There are also several open questions regarding proximate mechanisms, among them, the genetic and molecular bases of temperament traits and the longevity of early-life environmental effects. The least investigated issues addressed in this review were the ultimate or evolutionary mechanisms, which would reveal how cats’ interindividual differences in behavior originated and evolved.

The implications of cat temperament for the success of adoption, human–animal relationship, and welfare have been studied over the years. The roles of cat temperament and human personality in the success of adoption, owner satisfaction, and cat welfare have been reported. Temperament assessment also has the potential to offer insights that contribute to the development of cat-friendly strategies to minimize the impacts of cats’ predation on wildlife.

## Figures and Tables

**Table 1:** Summary of the 43 articles about temperament/personality in domestic cats included in this review. N = sample size and PC = principal component.

Reference	N	Environment	Method Description	Method Category	Main Dimensions
Feaver et al. [34]	14	Research facility	Behavioral observation and questionnaire	Coding/ Rating	Alert, sociable, equable
Turner et al. [52]	40	Research facility	Questionnaire	Rating	Friendliness
Adamec [62]	12	Research facility	Behavioral tests (response to rats and conspecific threat howls)	Coding	Aggressive, defensive
Reisner et al. [53]	13	Research facility	Behavioral tests (socialization and restraint)	Coding	Friendliness, defensive, aggression
McCune [20]	37	Research facility	Behavioral tests (familiar person, stranger, and novel object)	Coding	Boldness
Bradshaw and Cook [83]	36	Owned	Behavioral observation	Coding	Individual differences in post-feeding behaviors
Durr and Smith [78]	22	Owned	Behavioral observation and tests (novel stimulus (group and individual), approach to food, unfamiliar animals, and food competition)	Coding	Individual differences in social structure
Gosling and Bonnenburg [89]	440	Owned	Questionnaire	Rating	Big 5: Agreeableness, dominance, extraversion, impulsiveness, neuroticism
Lowe and Bradshaw [59]	29	Owned	Behavioral observation	Coding	Boldness
Siegford et al. [21]	20	Research facility	Feline Temperament Profile	Rating	Individual differences in positive and negative scores
Natoli et al. [27]	45	Free-ranging	Behavioral observation	Coding	Boldness
Lee et al. [92]	196	Owned	Questionnaire	Rating	PC1 (active, clever, curious, sociable), PC2 (emotional, friendly, protective), PC3 (aggressive, bad-tempered), PC4 (timid)
Zeigler-Hill and Highfill [93]	106	Owned	Questionnaire (Interpersonal Adjective Scales-Revised)	Rating	Dominance-submission, hostility-warmth
Iki et al. [67]	8	Research facility	Feline Temperament Profile	Rating	Individual differences in positive and negative scores
Marchei et al. [71]	82	Shelter	Behavioral tests (open-field and novel object)	Coding	Individual differences in the active-coping strategy and passive coping strategy
Wedl et al. [88]	40	Owned	Behavioral observation	Coding	Active, anxious, feeding, sociable, rough
Delgado et al. [76]	189	Owned	Questionnaire	Rating	Friendliness, aloofness, boldness, tolerance, trainability
Ramos et al. [68]	120	Owned	Questionnaire	Rating	Bossy, timid, easy going
Gartner and Powell [38]	100	Shelter	Questionnaire	Rating	Dominance, Impulsiveness, Neuroticism
Raihani [57]	14	Free-roaming	Behavioral observation	Coding	Individual differences in motor activity
Dickman and Newsome [103]	62	Owned	Behavioral observation and questionnaire	Coding/ Rating	Individual differences in hunting behavior and prey preference
Finkler and Terkel [82]	139	Free-ranging colonies	Behavioral tests (Novel object, Food bowl and Meat-in-the-box)	Coding	Boldness, Food dominance
Hudson et al. [24]	33	Research facility	Behavioral test (Separation)	Coding	Individual differences in separation calls and locomotion
Weiss et al. [101]	256	Shelter	Meet Your Match^®^ Feline-ality™ and Modified Meet Your Match^®^ Feline-ality™	Rating	Valiance, Independent-Gregarious
Arahori et al. [50]	94	Owned	Questionnaire	Rating	Openness, Friendliness, Roughness, Neuroticism
McDowell et al. [64]	90	Owned	Feline Temperament Profile and Cat Affect Temperament	Rating	Correlation between lateral bias and scale scores
Arahori et al. [49]	100	Owned	Questionnaire	Rating	Openness, Friendliness, Roughness, Neuroticism
Bennett et al. [94]	416	Owned	Questionnaire	Rating	Playfulness, Amiability, Dominance, Demandingness, Gullibility
Foster and Ijichi [72]	34	Shelter	Feline Temperament Profile	Rating	Individual differences in positive and negative scores
Ha and Ha [19]	251	Owned	Questionnaire	Rating	Cat social, Active, Human nonsocial, Human aggressive, Intense
Hudson et al. [56]	33	Research facility	Behavioral test (Separation)	Coding	Individual differences in separation calls
Litchfield et al. [9]	2802	Owned	Questionnaire	Rating	Big 5: Agreeableness, Dominance, Extraversion, Impulsiveness, Neuroticism
Rivera et al. [81]	41	Research facility	Behavioral tests (open-field and feline–human interaction)	Coding	Fearfulness
Menchetti et al. [96]	1270	Owned	Questionnaire	Rating	Sociability, reactivity, protectiveness, neuroticism, fearfulness
Evans et al. [8]	126	Owned	Questionnaire	Rating	Big 5: Agreeableness, dominance, extraversion, impulsiveness, neuroticism
Finka et al. [95]	3331	Owned	Questionnaire	Rating	Gregariousness, aggressiveness, aloofness/avoidance, anxiousness/fearfulness
Fukimoto [102]	71	Shelter	Modified Meet Your Match^®^ Feline-ality™	Rating	Agreeableness, openness, extraversion
Salonen et al. [51]	5726	Owned	Questionnaire	Rating	Aggression, extraversion, shyness
Stella and Croney [69]	55	Research facility	Behavioral observation and questionnaire	Coding/ Rating	Cluster 1 (shy, calm, mellow, and timid), Cluster 2 (active, playful, curious, and easygoing)
Urrutia et al. [79]	40	Shelter	Behavioral test (confinement in the carrier)	Coding	Individual differences in separation calls and motor activity
Vitale and Udell [80]	46	Owned/shelter	Behavioral tests (unknown person and owner interaction)	Coding	Sociability
Chacha et al. [106]	24	Owned	Behavioral test (quantity discrimination in a quasi-predatory situation)	Coding	Individual differences in prey preference
Fukimoto et al. [70]	53	Shelter	Modified Meet Your Match^®^ Feline-ality™	Rating	Agreeableness, openness, extraversion

## Supplementary Materials

The following are available online at https://www.mdpi.com/2076-2615/10/9/1516/s1: Table S1: Adjectives used in questionnaires for rating the assessment of cats’ temperament.
