# Supplementary material for: Temperament in Domestic Cats: A Review of Proximate Mechanisms, Methods of Assessment, Its Effects on Human—Cat Relationships, and One Welfare
Source: Animals (Basel). 2020 Aug 27;10(9):1516. doi: 10.3390/ani10091516 (PMC7552130; doi:10.3390/ani10091516)
Supplement: Supplementary file 1 [file animals-10-01516-s001.pdf]

animals

MDPI

Supplementary Files: Temperament in Domestic Cats: A Review of Proximate Mechanisms, Methods of Assessment, its Effects on Human—Cat Relationships, and One Welfare

Isadora de Castro Travnik [1,2], Daiana de Souza Machado [1,2], Luana da Silva Gonçalves [1], Maria Camila Ceballos [3] and Aline Cristina Sant'Anna [1,*]

Table S1. Adjectives used in questionnaires for the rating assessment of cats' temperament

References / Adjectives	Feaver et al. [34]	McDowell et al. [64]	Litchfield et al. [9]	Ha & Ha [19]	Bennet et al. [94]	Gosling & Bonnenburg [89]	Lee et al. [92]	Zeigler-Hil & Hightfill [93]	Gartner & Powell [38]	Delgado et al. [76]	Salonen et al. [51]	Stella et al. [116]	Arahori et al. [50]
Active	1	1	1	1			1		1	1		1	1
Activity level											1		
Energetic					1	1							
Agile	1			1									
Cunning								1					
Guick					1								
Quiet		1				1						1	
Calm			1		1				1	1		1	1
Relaxed					1	1							
Peaceful					1								
Confident		1			1								
Trusting			1						1				
Self-assured			1						1				
Persevering			1		1				1				
Persistent					1								
Decisive			1						1				
Curious	1		1	1	1		1		1			1	1
Excitable	1	1	1	1					1				1
Inquisitive			1										1
Watchful	1			1									
Vigilant			1						1				1
Attentive													1
Shy			1			1		1		1		1	
Shyness towards novel objects											1		
Shyness towards strangers											1		
Timid					1		1	1	1				1
Timid with strangers												1	
Constrained			1						1				
Submissive			1						1				
Bold			1			1				1			
Impulsive			1						1				1
Defiant			1										1
Unenvious						1							
Playful	1	1	1	1	1				1				1
Playful with people												1	
Playful with toys												1	

Sociable with people	1			1									
Social with familiar people												1	
Social with strangers												1	
Contact with people											1		
Friendly		1					1	1		1			1
Friendly towards people			1						1				
Affectionate		1	1						1				1
Friendly towards conspecifics									1				
Friendly towards other cats			1										
Sociable with cats	1			1									
Sociable							1						1
Likes attention but is not demanding												1	
Solitary	1		1	1					1				
Antisocial								1					
Distant								1					
Withdrawn						1							
Aloof										1			
Jealous			1			1			1				
Envious						1							
Stable			1						1				
Equable				1									
Equable with cats	1												
Aggressive	1	1		1	1		1						1
Aggressive towards people			1						1				
Hostile towards people	1			1									
Hostile towards cats	1			1									
Aggressive towards conspecifics									1				
Aggressive towards other cats			1										
Aggression towards strangers											1		
Aggression towards family members											1		
Aggression towards cats											1		
Tense	1		1	1					1				
Anxious			1			1			1				1
Nervous					1	1							1
Irritable			1										1

Fretful						1							
Rude						1							
Bad-tempered							1						
Harsh						1							
Cruel								1					
Ruthless								1					
Bossy													
Onwer-evaluated behavioural problem											1		
Fearful													1
Fearful of cats	1			1									
Fearful of other cats			1										
Fearful of conspecifics									1				
Fearful of people	1		1	1					1				
Vocal	1		1	1					1			1	
Verbal						1							
Talkative						1							
Untalkative						1							
Voracious	1			1									
Cooperative			1		1	1			1				
Obedient		1					1						
Trainable										1			
Smart			1						1				
Intellectual						1							
Clever							1						
Intelligent		1											
Unintellectual						1							
Unintelligent						1							
Independent		1			1		1		1			1	
Individualistic		1							1				
Excessive grooming											1		
Clean		1											
Wool sucking											1		
Suspicious			1						1				
Insecure			1						1				
Cautious					1								1
Calculating								1					
Apprehensive					1								
Dominant			1					1	1				1
Bullying			1						1				
Domineering					1								
Territorial					1								
Cool			1						1				
Warm					1	1							
Kind						1		1					
Emotional							1						
Charming					1								
Protective							1						
Gentle			1										1
Tender								1					
Outgoing								1					
Fairthful					1								

Sympathetic			1					
Boastless				1				
Extraverted			1					
Tolerant						1		
Undemanding				1				
Practical			1					
Prompt			1					
Considerate			1					
Efficient			1					
Easy going							1	
Stubborn						1		
Unsympathetic			1					
Mischievous		1						1
Intolerant						1		
Unkind			1					
Touchy			1					
Foolish		1						
Proud		1						
Stingy					1			
Cold			1					
Greedy	1							
Needy		1						
Needy, forces attention							1	
Demanding		1						
Imaginative			1					
Inventive	1							1
Creative			1					
Artistic			1					
Philosophical			1					
Aimless	1				1			
Erratic	1				1			
Eccentric	1				1			
Temperamental			1					
Moody	1		1					1
Systematic			1					
Organized			1					
Unsystematic			1					
Disorganized			1					
Uncreactive			1					
Forceful				1				
Loud		1						
Mellow							1	
Clumsy	1	1						
Careless			1					
Sloppy			1					
Inefficient			1					
Reckless	1				1			
Serius		1						
Gullible		1						
Confused		1						
Distractible	1				1			
Deliberate	1				1			
Quitting	1				1			1
Predictable	1				1			
Deep			1					
Bashful			1					
Complex			1					
Focused								1

Adaptable													1
Restless													1
**Total**	18	13	51	18	32	50	12	16	45	10	10	16	26
